# Ramucirumab Safety in East Asian Patients: A Meta-Analysis of Six Global, Randomized, Double-Blind, Placebo-Controlled, Phase III Clinical Trials

**DOI:** 10.1200/JGO.17.00227

**Published:** 2018-06-08

**Authors:** Chia-Jui Yen, Kei Muro, Tae-Won Kim, Masatoshi Kudo, Jin-Yuan Shih, Keun-Wook Lee, Yee Chao, Sang-We Kim, Kentaro Yamazaki, JooHyuk Sohn, Rebecca Cheng, Yawei Zhang, Polina Binder, Gu Mi, Mauro Orlando, Hyun Cheol Chung

**Affiliations:** **Chia-Jui Yen**, National Cheng Kung University Hospital, Tainan; **Jin-Yuan Shih**, National Taiwan University Hospital; **Yee Chao**, National Yang-Ming University and Taipei Veterans General Hospital; **Rebecca Cheng**, Eli Lilly and Company, Taipei, Taiwan; **Kei Muro**, Aichi Cancer Center Hospital, Nagoya; **Masatoshi Kudo**, Kindai University School of Medicine, Osaka-Sayama City, Osaka; **Kentaro Yamazaki**, Shizuoka Cancer Center, Shizuoka, Japan; **Tae-Won Kim** and **Sang-We Kim**, Asan Medical Center; **JooHyuk Sohn** and **Hyun Cheol Chung**, Yonsei University College of Medicine, Seoul; **Keun-Wook Lee**, Seoul National University College of Medicine, Seongnam, South Korea; **Yawei Zhang** and **Polina Binder**, Eli Lilly and Company, Bridgewater, NJ; **Gu Mi**, Eli Lilly and Company, Indianapolis, IN, USA; and **Mauro Orlando**, Eli Lilly and Company, Buenos Aires, Argentina.

## Abstract

**Purpose:**

Several ramucirumab trials have reported a higher incidence of selected adverse events (AEs) in East Asian (EA) patients with cancer versus non-EA patients. A meta-analysis was conducted across six completed phase III trials to establish the safety parameters of ramucirumab in EA compared with non-EA patients.

**Materials and Methods:**

Six global, randomized, double-blind, placebo-controlled, phase III registration trials investigating ramucirumab were assessed. Relative risks (RRs) and 95% CIs were calculated for selected all-grade and grade ≥ 3 AEs using fixed-effects and mixed-effects models. Ratio of RR and number needed to harm were calculated for AEs (all grade and grade ≥ 3) between EA and non-EA patients.

**Results:**

Of 4,996 randomly assigned patients receiving ramucirumab or placebo, 802 (16.1%) were EA (ramucirumab, n = 411; placebo, n = 391) and 4,194 were non-EA (ramucirumab, n = 2,337; placebo, n = 1,857). Patient baseline characteristics were generally balanced between treatment arms in EA and non-EA patients, excluding sex and body weight. Grade ≥ 3 AEs possibly associated with ramucirumab, which were increased in EA versus non-EA patients, included neutropenia (42.1% *v* 25.5%, respectively) and proteinuria (3.9% *v* 0.6%, respectively). There was an increase in the RR of several grade ≥ 3 AEs, including hypertension and proteinuria, in ramucirumab-treated EA and non-EA patients compared with placebo. The ratio of RR revealed no significant differences between EA and non-EA patients for all-grade and grade ≥ 3 AEs.

**Conclusion:**

Despite the enhanced propensity of selected AEs in EA patients relative to non-EA patients, there were no substantial differences in the RR for AEs possibly associated with ramucirumab in these phase III trials.

## INTRODUCTION

Ramucirumab is a human immunoglobulin G1 monoclonal antibody targeting vascular endothelial growth factor (VEGF) receptor-2,^1^ a key mediator of VEGF-induced angiogenesis.^2^ Six global, randomized, double-blind, placebo-controlled, phase III clinical trials have been completed, investigating ramucirumab in breast (ROSE),^3^ gastric (REGARD, RAINBOW),^4,5^ lung (REVEL),^6^ hepatocellular (REACH),^7^ and colorectal (RAISE)^8^ carcinomas.

Subsequently, ramucirumab (Cyramza; Eli Lilly, Indianapolis, IN) received worldwide and US Food and Drug Administration approval for gastric, lung, and colorectal cancers in the second-line setting.^9^ The safety parameters of ramucirumab across these six, global, phase III clinical trials have recently been investigated.^10^ This study, comprising a large patient population of 4,996, demonstrated a higher percentage of proteinuria, hypertension, low-grade bleeding, GI perforation, and wound-healing complications in ramucirumab-treated patients, consistent with antiangiogenic treatment. Notably, ramucirumab may be distinct among antiangiogenic agents in terms of no apparent increased risk of arterial thromboembolic events, venous thromboembolic events, high-grade bleeding, or high-grade GI bleeding.^10^

Subgroup analyses have been performed in selected phase III trials examining the efficacy and safety of ramucirumab in East Asian (EA) patients compared with non-EA patients.^11-14^ Overall, ramucirumab treatment conferred benefits to EA patients in terms of prolonging median survival times, improving progression-free survival, and increasing response rate.^11-13^ As for safety, EA patients have been reported to exhibit a higher incidence of certain adverse events (AEs) compared with non-EA patients.^3-8^ For instance, subgroup analyses from the RAINBOW and REVEL trials indicated higher incidence rates of any-grade neutropenia in ramucirumab-treated EA patients compared with those in the non-EA population (RAINBOW, 78% EA *v* 43% non-EA; REVEL, 84.4% EA *v* 53.4% non-EA).^5,6^

To further examine the safety of ramucirumab among EA patients, we conducted a meta-analysis examining the incidence of AEs possibly associated with VEGF-pathway inhibition in EA compared with non-EA patients across the six completed phase III trials. This analysis may assist and guide clinicians to optimize the treatment of EA patients with cancer with ramucirumab by maximizing efficacy while minimizing potential treatment-related toxicities.

## MATERIALS AND METHODS

Details of the study design and patients for each of the six randomized, double-blind, phase III ramucirumab trials have been published.^3-8^ A meta-analysis was conducted to review AEs in EA patients and non-EA patients across these six trials. The EA population was defined based on the geographic region in which patients enrolled at each study site. Each trial followed the guiding principles of the Declaration of Helsinki and the Good Clinical Practice Guidelines of the International Conference on Harmonization. All patients provided written informed consent. An overview of these trials is presented in Table 1.

**Table 1:**
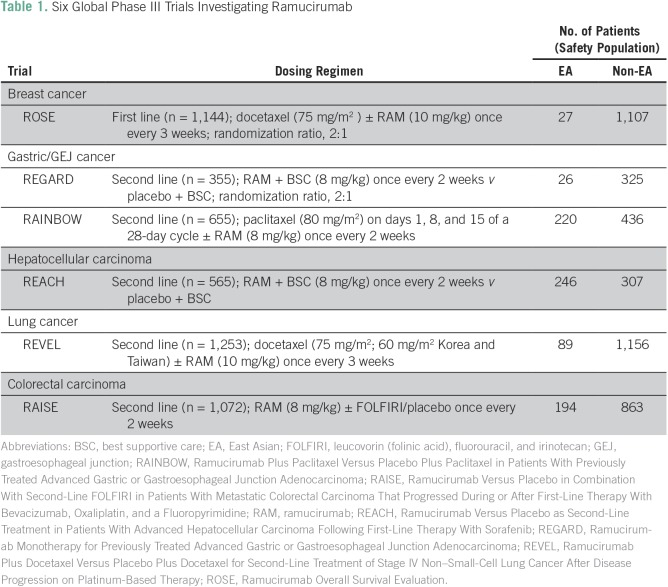
Six Global Phase III Trials Investigating Ramucirumab

AEs, identified via literature review to be possibly related to VEGF inhibition,^15^ were evaluated in the safety population for each trial. In addition, we report results for neutropenia, a common AE among EA patients. The safety population included all randomly assigned patients who received any dose of an investigational product (ie, ramucirumab or placebo). Grading of the AEs was based on Common Terminology Criteria for Adverse Events versions 3.0 to 4.02.

A key aspect of meta-analyses is to quantify the heterogeneity among a collection of studies.^16^ When there was no evidence of significant interstudy heterogeneity (Cochran’s *Q* test *P* > .05),^17^ the estimates of the relative risks (RRs) for each study were reported with 95% CIs using the fixed-effects (Mantel-Haenszel) method; otherwise, the random effects meta-analysis was adopted.^18^ The rmeta R package was used for computation (https://cran.r-project.org/web/packages/rmeta/index.html).^19^

The ratio of relative risk (RRR)^20^ was calculated to compare the two estimated RRs for each AE between EA and non-EA patients. An estimated RRR and the associated 95% CI were reported for each AE. There is no evidence of a difference in RRs if the 95% CI for the RRR contains 1.0. It should be noted that this test for interactions has limited power. The number needed to harm (NNH) was calculated for all-grade and grade ≥ 3 AEs using the following formula: 1/(risk of ramucirumab − risk of placebo).

## RESULTS

The safety population consisted of 4,996 patients randomly assigned to receive at least one dose of ramucirumab (n = 2,748) or placebo (n = 2,248). There were a total of 802 (16.1%) EA patients (ramucirumab, n = 411; placebo, n = 391) and 4,194 (83.9%) non-EA patients (ramucirumab, n = 2,337; placebo, n = 1,857). Patient baseline characteristics for EA and non-EA patients are summarized in Table 2. Baseline characteristics between EA and non-EA patients were generally comparable, with the exception of sex and body weight. Among EA patients, there was a higher percentage of male patients in both the ramucirumab and placebo treatment arms in comparison with non-EA patients (ramucirumab, 66.9% EA *v* 45.1% non-EA patients; placebo, 74.9% EA *v* 53.3% non-EA patients). In addition, the mean body weight of EA patients was less than that of non-EA patients (ramucirumab, 59.1 kg EA *v* 72.8 kg non-EA patients; placebo, 60.4 kg EA *v* 73.1 kg non-EA patients; Table 2).

**Table 2:**
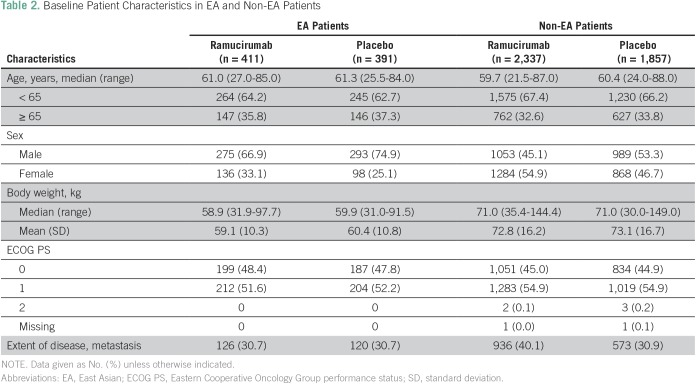
Baseline Patient Characteristics in EA and Non-EA Patients

The extent of treatment exposure for each of the six completed trials in EA and non-EA patients, including median duration of treatment and cumulative dose, is presented in Table 3. Median relative dose intensity of ramucirumab exposure was mostly similar between EA and non-EA patients with the exception of the RAISE study (79.7% EA *v* 89.2% non-EA patients).

**Table 3:**
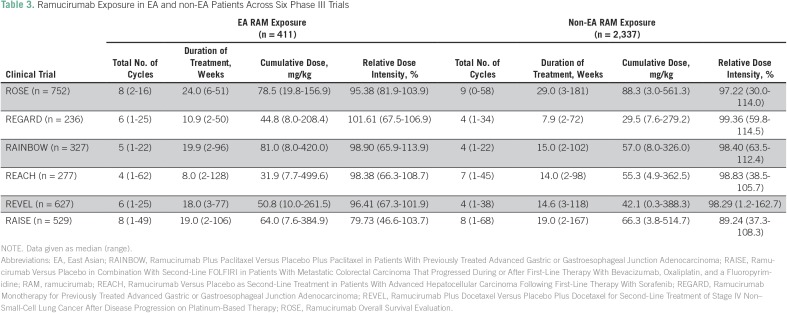
Ramucirumab Exposure in EA and non-EA Patients Across Six Phase III Trials

The incidence of AEs in EA and non-EA patients in completed phase III trials is listed in Table 4. In EA patients, AEs occurring in ≥ 10% of patients, regardless of grade, and at a higher rate in the ramucirumab-treated group versus the placebo-controlled counterpart, respectively, were hypertension (23.4% *v* 6.1%), proteinuria (24.6% *v* 7.7%), bleeding (41.8% *v* 18.9%), and neutropenia (53.0% *v* 36.6%). In non-EA patients, all-grade AEs occurring in ≥ 10% of patients and at a higher rate in the ramucirumab-treated group than the control group, respectively, included hypertension (20.9% *v* 7.7%), bleeding (36.8% *v* 19.0%), and neutropenia (33.2% *v* 29.6%). Among the grade **≥**3 AEs in Table 4, only neutropenia occurred in ≥ 10% of patients and at a higher rate in the ramucirumab-treated group than the control group, respectively, in EA patients (42.1% *v* 26.6%) and non-EA patients (25.5% *v* 20.5%).

**Table 4:**
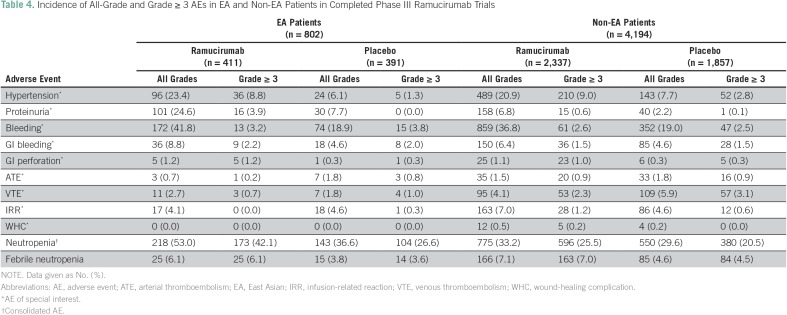
Incidence of All-Grade and Grade ≥ 3 AEs in EA and Non-EA Patients in Completed Phase III Ramucirumab Trials

In ramucirumab-treated patients, AEs occurring with at least a 5% incidence difference between EA and non-EA patients were all-grade proteinuria (24.6% EA *v* 6.8% non-EA patients), bleeding (41.8% EA *v* 36.8% non-EA patients), and neutropenia (53.0% EA *v* 33.2% non-EA patients). Neutropenia was the only grade ≥ 3 AE with a ≥ 5% incidence difference between EA and non-EA patients (42.1% EA *v* 25.5% non-EA patients).

The RR and corresponding RRR of AEs in EA and non-EA patients are listed in Table 5. In cases where the Cochran’s *Q* test *P* < .05, a random-effects model was adopted (instead of a fixed-effects model) to accommodate for the interstudy variability; in Table 5, the RR is marked with a '#' for such cases. In EA patients, adding ramucirumab was associated with increased risk of all-grade hypertension (RR, 3.6; 95% CI, 2.4 to 5.5), proteinuria (RR, 3.1; 95% CI, 2.2 to 4.5), bleeding (RR, 2.2; 95% CI, 1.8 to 2.8), GI bleeding (RR, 1.9; 95% CI, 1.1 to 3.2), and neutropenia (RR, 1.5; 95% CI, 1.3 to 1.7). In non-EA patients, adding ramucirumab was associated with an increased risk of all-grade hypertension (RR, 2.6; 95% CI, 2.2 to 3.1), proteinuria (RR, 3.4; 95% CI, 2.4 to 4.7), bleeding (RR, 1.9; 95% CI, 1.7 to 2.1), GI bleeding (RR, 1.5; 95% CI, 1.2 to 2.0), GI perforation (RR, 3.0; 95% CI, 1.3 to 6.9), neutropenia (RR, 1.3; 95% CI, 1.1 to 1.6), and febrile neutropenia (RR, 1.6; 95% CI, 1.2 to 2.1). For several AEs, the NNH differed between EA and non-EA patients (Table 5). According to our NNH calculations, EA patients exhibited an absolute increased risk of all-grade proteinuria (one in six EA *v* one in 22 non-EA patients), GI bleeding (one in 24 EA *v* one in 54 non-EA patients), GI perforation (one in 104 EA *v* one in 134 non-EA patients), and neutropenia (one in six EA *v* one in 28 non-EA patients).

**Table 5:**
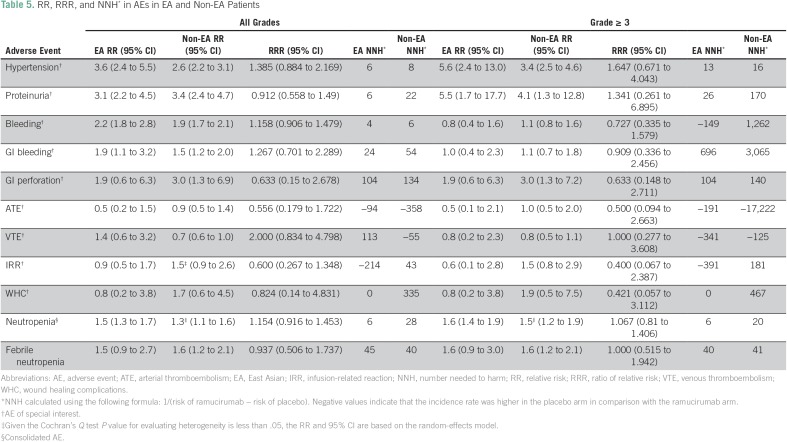
RR, RRR, and NNH* in AEs in EA and Non-EA Patients

There was an increase in the RR of several grade ≥ 3 AEs in EA patients, including hypertension (RR, 5.6; 95% CI, 2.4 to 13.0), proteinuria (RR, 5.5; 95% CI, 1.7 to 17.7), and neutropenia (RR, 1.6; 95% CI, 1.4 to 1.9); and an increase in RR for hypertension (3.4; 95% CI, 2.5 to 4.6), proteinuria (RR, 4.1; 95% CI, 1.3 to 12.8), GI perforation (RR, 3.0; 95% CI, 1.3 to 7.2), neutropenia (RR, 1.5; 95% CI, 1.2 to 1.9), and febrile neutropenia (RR, 1.6; 95% CI, 1.2 to 2.1) in non-EA patients (Table 5). EA patients also exhibited an absolute increased risk in the NNH of grade ≥ 3 AEs, including proteinuria (one in 26 EA *v* one in 170 non-EA patients), GI bleeding (one in 696 EA *v* one in 3,065 non-EA patients), GI perforation (one in 104 EA *v* one in 140 non-EA patients), and neutropenia (one in six EA *v* one in 20 non-EA patients; Table 5). No substantial differences in the NNH were observed between EA and non-EA patients in terms of grade ≥ 3 febrile neutropenia (one in 40 EA *v* one in 41 non-EA patients) and hypertension (one in 13 EA *v* one in 16 non-EA patients). The RRR revealed no significant differences between EA and non-EA patients for all-grade and grade ≥ 3 AEs (Table 5; Data Supplement).

Analysis of RRs in ramucirumab plus chemotherapy combination trials (ie, ROSE, RAINBOW, RAISE, REVEL) revealed an increase in the risk of developing all-grade and grade ≥ 3 proteinuria and GI perforation (Data Supplement). Equivalent analysis of ramucirumab monotherapy trials (ie, REGARD, REACH) indicates an increased risk of all-grade hypertension, proteinuria, bleeding, and neutropenia, as well as increased risk of grade ≥ 3 hypertension (Data Supplement). Overall, the RR of selected AEs was mostly comparable between EA and non-EA patients in both ramucirumab combination and monotherapy cohorts (Data Supplement).

## DISCUSSION

Ramucirumab, like other VEGF-targeted treatments, is associated with several “classes” of AEs. These AEs have been well documented and encompass hematologic and cardiovascular toxicities.^3-6,8,21,22^ It has been increasingly reported that EA patients may have greater toxicity to chemotherapy and targeted therapies compared with Western patients.^23,24^ For this reason, subanalyses are often conducted in EA patients with the aim of confirming that a regimen with a positive risk-benefit profile in a global population also confers meaningful efficacy with an acceptable safety profile in the EA subpopulation. We describe a meta-analysis of six completed phase III ramucirumab trials to explore whether EA patients are at increased risk of AEs associated with ramucirumab therapy. Differences were noted in the incidence rates of selected AEs between EA and non-EA patients; however, based on comparative exposure data, these differences did not jeopardize ramucirumab treatment and patients were able to continue therapy.

The EA patient cohort across all six phase III ramucirumab trials exhibited comparable baseline characteristics in comparison with non-EA patients, with the exception of sex (more male patients in the EA patient cohort) and body weight (EA patients weighed less). The sex imbalance may be due to the relatively low number of EA patients enrolled in the ROSE breast cancer trial (n = 27). Despite EA patients having a lower body weight compared with non-EA patients, ramucirumab exposure was mostly comparable between these patient cohorts. Although we observed variations in the number of ramucirumab treatment cycles and in median cumulative doses between trials, which may affect the frequency and grade of AEs, the overall dose intensity was mostly comparable between EA and non-EA patients.

Hypertension is a frequently observed AE associated with VEGF inhibitors^21^ and is commonly reported in ramucirumab clinical trials.^4-8^ Our analysis revealed no obvious differences in the risk of hypertension in the ramucirumab arm between EA and non-EA patients (23% EA *v* 21% non-EA patients). Although there was an increased trend in RR for grade ≥ 3 hypertension (5.6 EA *v* 3.4 non-EA patients), our findings suggest that the risk of grade ≥ 3 hypertension is low under antihypertensive intervention and comparable between EA and non-EA patients.

Proteinuria is a known AE occurring frequently in patients receiving anti-VEGF therapy, because of the suppression of nephrin, an important protein for the maintenance of the glomerular slit diaphragm.^25^ Our findings indicate that treatment with ramucirumab increases the risk of proteinuria in EA patients with cancer; however, proteinuria overall was of low-grade severity and did not lead to treatment discontinuation. Notably, the incidence of proteinuria in placebo-treated EA patients was also higher relative to their non-EA counterparts; therefore, the RRs were similar between EA and non-EA patients and the RRR was not significant. Some studies have reported that Asian patients are more vulnerable to developing proteinuria in comparison with Western patients.^11,26,27^ Given that we did not evaluate confounding factors, such as concomitant use of nephrotoxic agents or previous cisplatin exposure in GI cancers, the reasons behind ethnic differences in absolute incidence of proteinuria are far from being understood. Because proteinuria is a risk factor for cardiovascular disease and loss of renal function, periodic monitoring of urinary protein and appropriate intervention should be recommended for all ramucirumab-treated patients.

Neutropenia incidence was increased in EA patients in comparison with non-EA patients, but the incidence of febrile neutropenia was low and similar between EA and non-EA patients. Given that the increase in neutropenia incidence in EA patients was mainly noted in ramucirumab-chemotherapy combination trials, it is possible that this observed increase was driven by chemotherapies. Support for this conclusion comes from the improvement in the safety profile among EA patients accompanied by a dose reduction of docetaxel in the REVEL trial.^6^ The decrement of docetaxel starting dose from 75 mg/m^2^ to 60 mg/m^2^ in EA patients reduced the incidence of neutropenia and febrile neutropenia to a rate similar to that observed for non-EA patients.^13^

Consistent with the intent-to-treat populations, all-grade bleeding was increased in ramucirumab-treated patients in comparison with placebo-treated patients, and this was observed in EA and non-EA patients. Importantly, the incidence of grade ≥ 3 bleeding was low and similar in both treatment arms and between EA and non-EA patients. The incidences of additional grade ≥ 3 AEs associated with VEGF-targeted treatments were also low and comparable between EA and non-EA patients. These include GI bleeding and GI perforation, arterial and venous thromboembolism, and wound-healing complications. Our meta-analysis suggests that ramucirumab is well tolerated in EA patients using the dosage and regimen outlined in the six completed phase III trials under investigation.

The NNH provides a useful indication to clinicians and patients of the absolute risks involved with treatment. Although no substantial differences were observed in the RR of AEs between EA and non-EA patients, the incidence rates (and rate differences) of selected AEs were increased in EA patients, including proteinuria, neutropenia, and bleeding. The NNH supports this finding, providing clinicians with an evidence-based tool to assist with treatment decisions regarding optimal supportive care and dose modification concerning EA patients with cancer.

To the best of our knowledge, this is the first and largest individual-patient meta-analysis to evaluate the safety profile of ramucirumab among East Asian patients with cancer. In this meta-analysis, we found an increased RR in certain AEs in EA patients. However, the results of this analysis should be interpreted with caution: there were limited numbers of EA patients in some trials, and patients were categorized by their geographic location, and limited ethnicity data were available. In addition, heterogeneity between studies should be noted in terms of cancer types, treatment regimens including the trial chemotherapy backbone, and patient characteristics. Furthermore, wide confidence intervals were observed, which reflect substantial uncertainty in the point estimation of RR for some AEs. No obvious differences in RRR were noted between EA and non-EA patients; however, this interaction test may not be powerful enough to detect a significant difference.^20^ As with many clinical trials, patients enrolled in ramucirumab clinical trials may not represent patients in the general population, because trial patients are screened for adequate organ function and concurrent morbidities and medications.

Benefit versus risk is an important factor for clinicians and patients when making decisions concerning cancer treatments. Collectively, results from our meta-analysis were consistent with the overall ramucirumab safety profile^10^ as well as demonstrating that ramucirumab has a similar risk profile in EA patients compared with non-EA patients enrolled in clinical trials. In addition to routine clinical practices, clinicians should monitor patients for potential ramucirumab-related AEs, including hypertension and proteinuria. The risks associated with ramucirumab may be increased by other factors, including patient comorbidities and concomitant medications, prior therapies, and tumor characteristics.

Across these six completed phase III ramucirumab trials, the majority of AEs discussed here in EA patients were manageable and did not jeopardize EA patients’ cancer therapy. Patients were able to continue to receive ramucirumab therapy to achieve maximum clinical benefits.
